# Low-value medical orders: bridging the gap between concept and action in reducing healthcare waste

**DOI:** 10.3389/fpubh.2026.1793477

**Published:** 2026-03-03

**Authors:** Jin Wen, Wenjuan Tao, Xiru Yu

**Affiliations:** 1 Institute of Hospital Management, West China Hospital, Sichuan University, Chengdu, China; 2 School of Biomedical Engineering, Tsinghua University, Beijing, China

**Keywords:** clinical decision support, de-implementation, low-value care, low-value medical orders, value-based healthcare

## Abstract

Healthcare systems worldwide face the dual challenge of improving quality while controlling costs. Value-based healthcare has emerged as a guiding framework, yet a substantial proportion of delivered services remain low-value, providing minimal benefit while consuming resources and potentially causing harm. While low-value care has been extensively studied at the service level, less attention has been paid to the fundamental unit that initiates all healthcare delivery: the medical order. This commentary introduces the concept of low-value medical orders (LVMO) as a complementary framework to existing low-value care paradigms. By focusing on the point of clinical decision-making, LVMO offers a more actionable target for real-time intervention. We distinguish LVMO from low-value care across multiple dimensions and discuss how this framework can inform multilevel strategies combining clinician-focused interventions with broader system reforms. Drawing on recent systematic reviews of de-implementation interventions, we identify critical research gaps and propose directions for developing effective, sustainable approaches to reduce unnecessary ordering.

## Introduction

Value-based healthcare (VBHC), first defined by Porter and Teisberg, is emerging as a leading model in modern health systems, reframing healthcare delivery around the goal of maximizing patient outcomes per unit of cost (1). It shifts focus from volume to value, emphasizing long-term outcomes that matter to patients, efficiency, and integrated care. VBHC has been adopted globally as a strategy to combat unsustainable spending while improving quality. For example, the World Health Organization, in collaboration with the Chinese government, endorsed VBHC as a means to integrate prevention, treatment, and personalized care (2). However, critics have noted that current VBHC models do not always adequately incorporate patient-centered outcomes, and implementation varies considerably across settings (3).

Despite this paradigm shift, healthcare waste remains pervasive. The Organization for Economic Co-operation and Development (OECD) estimates that up to 20% of health spending is wasted on ineffective, low-value, or unnecessary care (4). In the United States, wasteful spending is estimated between $760 billion and $935 billion annually, including unnecessary services, administrative inefficiencies, and missed prevention opportunities (5). This waste not only strains resources but also exposes patients to potential harm from invasive procedures, diagnostic cascades, and psychological distress.

## From low-value care to low-value medical orders

Low-value care (LVC) refers to medical services that offer minimal or no clinical benefit relative to their cost or risk. Examples include unnecessary imaging for uncomplicated low back pain, routine preoperative testing in low-risk surgeries, and continued use of obsolete diagnostics. Such care is driven by multiple factors: defensive medicine, habit, patient demand, system defaults, and misaligned incentives (6–11). Several methods have been developed to identify LVC, including claims-based measures (7), guideline-driven recommendations such as the Choosing Wisely campaign, and electronic health record (EHR) data (12). However, these approaches are often designed for population-level analysis and lack granularity for individual patient identification. They reveal patterns of overuse but frequently fail to enable direct intervention at the point of care.

Each medical action, whether an imaging examination, a laboratory test, medication prescription, or referral, is initiated by a physician's order. This means that virtually all healthcare delivery and associated costs are order-driven. Based on this logic, whether a medical order has patient value deserves attention as a distinct concept from LVC. We therefore propose low-value medical orders (LVMO) as a complementary framework. The value of a medical order should be assessed along the “disease–medical order–outcome pathway”: for a given disease or clinical condition, an order is considered low-value when the expected health outcomes do not sufficiently justify the associated costs and potential risks. Operationally, this includes tests, procedures, or consultations ordered outside of evidence-based guidelines or without specific clinical indication, where potential harm or resource waste outweighs the likelihood of meaningful clinical benefit.

Table 1 illustrates the key distinctions between LVC and LVMO across multiple dimensions. While LVC operates at the service and outcome level, LVMO focuses on the decision and behavior level. This distinction has important practical implications: LVC is typically identified retrospectively through claims analysis, whereas LVMO can potentially be identified and addressed in real-time at the point of care. Furthermore, while LVC attribution is often diffuse across multiple stakeholders, LVMO can be directly traced to the ordering clinician, enabling more targeted feedback and intervention.

**Table 1:** Comparison of low-value care (LVC) and low-value medical order (LVMO).

**Dimension**	**Low-Value Care (LVC)**	**Low-Value Medical Order (LVMO)**
Definition	Medical services, tests, or treatments that provide little or no clinical benefit relative to their cost, risk, or resource consumption	Tests, procedures, or consultations ordered without specific clinical indication or outside guideline recommendations, where potential harm or resource waste outweighs expected benefit
Conceptual Level	Service and outcome level	Decision and behavior level
Unit of Analysis	Healthcare service event	Individual order
Timing of Identification	Mostly retrospective (*post-hoc* analysis)	Real-time identification possible (prospective or concurrent intervention)
Intervention Target	Multiple levels: system, policy, payment, culture	Point-of-care clinical decision-making
Measurement Methods	Claims data, guideline adherence assessment	Electronic health record order data, clinical decision support system triggers
Attribution	Multifactorial, shared responsibility across stakeholders	Directly traceable to the ordering physician
Typical Example	A low-risk patient received unnecessary preoperative cardiac evaluation	Ordering a cardiology consultation for an ASA I patient undergoing minor elective surgery

Note: ASA, American Society of Anesthesiologists physical status classification.

It is important to note that, because the LVMO framework should define order value within the “disease–medical order–outcome” pathway, outcome considerations are inherently embedded at the point of decision-making. Nevertheless, in practice, the alignment between order-level decisions and outcome-level value is not always straightforward. In some cases, orders that appear guideline-concordant at the time of decision-making may contribute to low-value outcomes through mechanisms such as diagnostic cascades, in which initially appropriate tests trigger a series of follow-up investigations with diminishing marginal benefit; context-dependent variation, where guideline-concordant orders yield suboptimal outcomes in specific patient subgroups; and system-level factors, including fragmented care coordination, that may render individually appropriate orders collectively redundant. Recognizing this complexity reinforces that the LVMO framework is designed to complement, not replace, outcome-level evaluation. Effective reduction of healthcare waste requires integrated monitoring at both the decision level and the outcome level, with iterative feedback to continuously refine ordering criteria.

## Common types of low-value medical orders

Common types of LVMO include routine or repetitive orders without clinical change, such as daily laboratory tests in stable hospitalized patients; obsolete or misused tests, for example, using creatine kinase-MB (CK-MB) instead of troponin for acute coronary syndrome evaluation (13); duplicate testing within short timeframes; inappropriate referrals, such as seeking cardiology clearance for low-risk surgery (14); and system-driven orders, including default order sets applied without individual patient reassessment. These orders are not merely inefficient; they can directly harm patients through false positives, invasive follow-up procedures, radiation exposure, and psychological distress. Moreover, they divert resources from high-value care.

The magnitude of unnecessary ordering is substantial. Studies estimate that up to 30% of laboratory tests may be unnecessary (5), inappropriate imaging accounts for billions in wasteful spending annually (15), and unnecessary specialist referrals delay care while increasing costs (16). These figures underscore the potential impact of systematically addressing LVMO.

## A multilevel approach to reducing low-value orders

While the LVMO framework emphasizes the clinician's role as the originator of orders, we recognize that effective and sustainable change requires multilevel interventions. As others have noted, combining “top-down” policy and organizational changes with “bottom-up” clinician engagement, and balancing incentives (“carrots”) with accountability mechanisms (“sticks”), is most likely to achieve meaningful and sustained impact (17–19). The determinants of LVC and LVMO are interconnected across social, knowledge, and system levels, and no single intervention is likely to be sufficient (20).

Recent systematic reviews provide valuable guidance on intervention effectiveness. Cliff et al. (21) reviewed Choosing Wisely interventions and found that multicomponent strategies combining education, feedback, and system changes were more effective than single-component approaches. Heus et al. (22) similarly found that interventions targeting multiple levels showed greater promise for sustained de-implementation—the systematic process of reducing or abandoning low-value clinical practices. However, both reviews noted significant heterogeneity in intervention design and outcomes measurement, highlighting the need for more standardized approaches.

## Clinician-level interventions

Clinical decision support (CDS) systems that embed guidelines into computerized physician order entry (CPOE) systems have shown promise in reducing duplicate testing and obsolete orders (23). These systems can provide real-time alerts, suggest alternatives, or require justification for potentially low-value orders. However, alert fatigue remains a significant challenge, and the optimal design of CDS interventions requires further investigation.

Audit and feedback, which informs physicians about their ordering patterns compared to peers, can reduce cost and variability (24). The effectiveness of feedback depends on its timeliness, specificity, and actionability. Providing individualized reports with concrete recommendations appears more effective than aggregate data alone.

Educational interventions, particularly those targeting trainees, can instill value-conscious ordering habits early in clinical practice. Engaging clinical champions who model appropriate ordering behavior may amplify these effects. Behavioral economic approaches, including nudges and choice architecture modifications, offer additional tools for influencing ordering decisions without restricting physician autonomy (25).

## System and policy-level interventions

System redesign strategies include revising default order sets to remove low-value options, implementing minimum retest intervals, establishing laboratory formulary committees, and expanding e-consult programs to reduce unnecessary in-person specialist referrals (26, 27). These approaches modify the choice environment to make high-value ordering the path of least resistance.

Payment reform represents a critical policy lever. Comparison of payment changes and Choosing Wisely recommendations in the United States and Canada demonstrates that financial incentives can significantly influence ordering behavior, though effects vary by test type and clinical context (28). Aligning reimbursement with value-based ordering metrics, while complex to implement, may provide sustained motivation for change.

Importantly, in many healthcare systems, prevailing fee-for-service models may inadvertently incentivize higher ordering volumes, as physician or institutional revenue is tied to the quantity rather than the appropriateness of services delivered. This creates a fundamental misalignment between financial incentives and value-based ordering. Addressing this requires not only reforming payment structures but also introducing positive incentives for physicians who demonstrate judicious ordering practices. Examples include shared savings programs that reward departments or clinicians for reducing unnecessary orders without compromising patient outcomes, performance-based bonuses tied to adherence to evidence-based ordering guidelines, and public recognition programs that highlight exemplary practice. Conversely, the absence of such incentives—or the persistence of volume-based rewards—may undermine even the most well-designed clinical decision support systems and educational interventions.

Organizational culture and leadership commitment are essential enabling factors. Healthcare systems that explicitly prioritize high-value care and provide infrastructure support for improvement initiatives create environments where clinician-level interventions are more likely to succeed.

## Research gaps and future directions

Several important research gaps remain. First, while methods for measuring LVC at the population level are well-established (29), validated approaches for identifying LVMO at the individual patient and clinician level require further development. Real-time EHR-based identification systems hold promise but need rigorous evaluation.

Second, the optimal design of point-of-care interventions remains unclear. Questions persist regarding the ideal intensity of CDS alerts (hard stops vs. soft stops), the most effective feedback formats, and how to balance intervention effectiveness against workflow disruption and alert fatigue.

Third, implementation science frameworks should be applied more systematically to de-implementation research. Understanding context-specific barriers and facilitators, and adapting interventions accordingly, is critical for achieving sustained change across diverse clinical settings (20).

Fourth, patient engagement in reducing LVMO deserves greater attention. While Choosing Wisely and similar campaigns have developed patient-facing materials, evidence on their effectiveness in changing ordering patterns through patient-clinician conversations remains limited (30).

## Conclusion

Low-value medical orders represent a critical and often overlooked aspect of healthcare value, situated at the intersection of physician behavior, system design, and patient outcomes. The LVMO framework complements existing LVC paradigms by focusing attention on the fundamental unit of clinical action: the order. This focus enables real-time identification and intervention, direct attribution for feedback purposes, and integration with existing EHR and CDS infrastructure.

Addressing LVMO effectively requires multilevel strategies that combine clinician-focused interventions with organizational and policy reforms. Future efforts should prioritize developing systems to track low-value orders at the clinician level, optimizing smart and patient-specific alerts within electronic health records, aligning incentives with value-based ordering metrics, providing continuous education on the clinical and economic implications of ordering decisions, and investing in research to identify the most effective de-implementation approaches across diverse contexts.

Reducing low-value ordering is unlikely to be fully achievable, but meaningful progress is possible. Ultimately, improving the quality and value of healthcare starts with improving the quality and value of medical orders. By focusing on this fundamental unit of care while attending to the broader system factors that shape ordering behavior, we can reduce waste, enhance patient safety, and move closer to the ideals of value-based healthcare.

## Data Availability

The original contributions presented in the study are included in the article/supplementary material, further inquiries can be directed to the corresponding author.
